# Restricting movements of lower face leaves recognition of emotional vocalizations intact but introduces a valence positivity bias

**DOI:** 10.1038/s41598-022-18888-0

**Published:** 2022-09-27

**Authors:** Kinga Wołoszyn, Mateusz Hohol, Michał Kuniecki, Piotr Winkielman

**Affiliations:** 1 Institute of Psychology, Jagiellonian University, Kraków, Poland; 2 Copernicus Center for Interdisciplinary Studies, Jagiellonian University, Kraków, Poland; 3 Department of Psychology, University of California San Diego, La Jolla, USA

**Keywords:** Psychology, Human behaviour

## Abstract

Blocking facial mimicry can disrupt recognition of emotion stimuli. Many previous studies have focused on facial expressions, and it remains unclear whether this generalises to other types of emotional expressions. Furthermore, by emphasizing categorical recognition judgments, previous studies neglected the role of mimicry in other processing stages, including dimensional (valence and arousal) evaluations. In the study presented herein, we addressed both issues by asking participants to listen to brief non-verbal vocalizations of four emotion categories (anger, disgust, fear, happiness) and neutral sounds under two conditions. One of the conditions included blocking facial mimicry by creating constant tension on the lower face muscles, in the other condition facial muscles remained relaxed. After each stimulus presentation, participants evaluated sounds’ category, valence, and arousal. Although the blocking manipulation did not influence emotion recognition, it led to higher valence ratings in a non-category-specific manner, including neutral sounds. Our findings suggest that somatosensory and motor feedback play a role in the evaluation of affect vocalizations, perhaps introducing a directional bias. This distinction between stimulus recognition, stimulus categorization, and stimulus evaluation is important for understanding what cognitive and emotional processing stages involve somatosensory and motor processes.

## Introduction

Dozens of behavioral studies have consistently corroborated Adam Smith’s^1^ insight that human beings tend to mimic, or imitate, observed behavior. This phenomenon includes body postures^2^, hand gestures^3^, head movements^4^, voice parameters^5^, pupils^6^, and facial expressions^7,8^. Moreover, because the execution of a congruent behavior facilitates recognition of the perceived one^9–13^, the current understanding of motor imitation suggests that it extends beyond being a simple by-product of stimulus–response associations^14^.

Here, we focus on facial mimicry; namely, the tendency to spontaneously imitate facial expressions of other people^7,15–17^. Although in most cases, facial mimicry is too weak to be detected by the naked eye, numerous electromyographic studies have demonstrated that even passively observing facial expressions of emotions can activate the observers’ relevant muscles in an automated, rapid, and emotion-specific manner^18,19^.

Importantly, the large body of research employing causal paradigms revealed that blocking spontaneous facial activity disrupts recognition of emotional categories displayed on perceived faces, which suggests, at least, a facilitative role of imitation^20–24^. Recently, the effect of blocking facial expressions has been shown to be generalised to the recognition of body expressions^25^. The results of these causal interventions are predominantly interpreted in line with the sensorimotor simulation account^26,27^ that emerged within embodied psychology^28,29^. However, since the existing body of evidence comes predominantly from tasks with visually displayed emotions and focused on categorical judgements, our knowledge of the emotions expressed through other than visual channels remain scarce. Thus, the main purpose of the present study was to investigate the causal involvement of facial mimicry in recognizing emotional categories of human affect vocalizations, their valence, and arousal.

### Embodiment and simulation

Over the last four decades, numerous researchers have extensively argued that the body (including its brain representation) both constrains and facilitates cognition. As a result, higher cognition has begun to be framed as at least partially grounded in sensorimotor activity^30–32^. This view is popular in many fields of psychological science, including social cognition^28,29^. Although the scope of the embodiment^33–38^, and its methodological commitments^39–42^ are under extensive discussion, numerous researchers in the field agree that at least some aspects of conceptual understanding rely on a mechanism of simulation^26,27,43^. These simulations involve reenactments of sensorimotor and somatosensory networks of the brain that are primarily active during physical interactions with exemplars of a given category. Notably, proponents of embodied cognition argue that spontaneous imitation, or automatic mimicry, can reflect sensorimotor simulation, and thus, is involved in the conceptual processing of emotion^28,44,45^.

Various studies have demonstrated that spontaneous mimicry plays a causal role in face processing^44^. Specifically, these studies have focused on how manipulating the activity of facial muscles or somatosensory areas influences the recognition of several aspects of emotional expressions. For example, the results of the study with the “pen procedure” by Oberman, Winkielman, and Ramchandran^20^ revealed that manipulating the muscular activity of the lower part of the face (holding a pen with the teeth) selectively impaired recognition of happy and disgusted emotional faces; emotions that typically strongly activate the muscles affected by the procedure. Along similar lines, Ponari and colleagues^23^ also found hindered recognition of facial expressions of happiness and disgust during the increased tonic activity of lower face muscles (holding chopsticks with the teeth) and the impairment of the recognition of angry facial expressions while the brow area was activated (﻿drawing together two little stickers placed close to the inner edge of eyebrows). Judgments of fear expressions were influenced independently of which area of the perceiver’s face was manipulated.

The selectivity of the mimicry blocking effect is also an argument against the explanation that observed reduction in accuracy rate is merely due to increased cognitive load in blocking conditions. Corroborating results have also been obtained in other behavioral studies^22,24,44,46^. The role of sensorimotor processes in face perception has also been examined in studies on the effects of brain damage or its temporal deactivation in laboratory conditions. Both lesions within the sensorimotor areas^47^ and transitory inactivation of the somatosensory face-related regions by repetitive TMS disrupted face recognition^48^ and judging whether a presented smile reflected genuine amusement or not^49^.

Importantly, Borgomaneri and colleagues^25^ investigated whether the effect of disrupting facial mimicry goes beyond hindering the recognition of facial expressions. They found that blocking facial mimicry (“pen procedure”) in perceivers disturbs not only their recognition of happiness portrayed on faces but also expressed with whole-body postures. It suggests that facial mimicry is not engaged solely in processing others’ facial expressions but is a part of the conceptual processing of emotion. This is consistent with the study by Connolly and colleagues^50^, who showed that, instead of multiple domain-specific factors, there is a supramodal emotion recognition ability that is linked to the recognition of both facial and bodily expressions. They demonstrated that this ability also generalises across modalities and is linked to the recognition of emotional vocalizations. The latter has been also suggested by Hawk and colleagues^51^, who explored the effect of cross-channel mimicry. They tested the impact of facial responses on the processing of emotional human vocalizations. The study presented participants with sounds that gradually transitioned from laughing to crying and vice versa. The participants’ task was to detect the moment of change of expression while holding the pen in one of two ways: in their teeth or in their hands. The results were contradictory to earlier studies using visual stimuli, reporting poorer or slower performance with mimicry blocking manipulations^21^. Specifically, the study by Hawk and colleagues revealed that the inhibition of spontaneous facial responses sped up the detection of change, as compared to when facial movements were not blocked. The authors interpreted this finding by suggesting that in a default, uninhibited condition, cross-channel mimicry of heard expressions strengthens the focus of attention on, and engagement with, the mimicked expression, which typically leads to slower responses when an opposite emotion appears.

Overall, the above results of causal interventions offer a persuasive argument against the view that sensorimotor activations during perception of emotional stimuli are just a by-product with no causal role. On the one hand, our knowledge regarding the generality of sensorimotor simulation as a mechanism supporting emotion processing remains limited because the discussed studies predominantly employed visual stimuli. On the other hand, the above-summarized studies primarily investigated the recognition of distinct emotion categories. While there is a growing consensus that valence and arousal are more fundamental dimensions of emotions than such categories^52–54^, they are still understudied in the embodied simulation account. The first of these dimensions, valence (negative–positive), is interpreted as a qualitative aspect of emotion, while the latter, arousal, as its intensity (low–high). In a nutshell, the approach defended by Russell and Barrett^54^ assumes that the emotion has a core component that is sufficiently defined by its valence and arousal dimensions, as well as additional elements, influenced by particular circumstances, which includes appraisals, behavioural reactions, the categorization of the experienced emotion, etc. Hence the dimensional aspect of emotion is more fundamental and requires less cognitive operations in juxtaposition to emotional categorization and conceptual processing. Additionally, this theoretical approach is uniquely useful for research involving physiological measurement as the elements of the core component, namely, arousal and valence dimensions, were shown to have very reliable physiological correlates^55,56^.

The study that addressed the issue of the impact of facial expression on valence and arousal ratings was conducted by Hyniewska and Sato^57^ in the context of the facial feedback hypothesis. They found that when participants activated the *zygomaticus major* following the instruction (“raise the cheeks”), they rated emotional facial expressions, both static and dynamic, as more positive compared to activating the *corrugator supercilii* (“lower the brows”). Consistently, many previous studies on facial feedback have shown that producing relevant facial movements may also impact various aspects of cognitive processing, including affective evaluation of emotional material^58–61^, memory of valence-consistent stimuli^62^, perceived valence of observed facial expressions^57^, and their intensity^63^. Moreover, the very act of producing facial expressions has been shown to evoke a corresponding emotional experience, including physiological reactions similar to actual emotions^64^. As summarized in a recent review, the effects of such facial feedback manipulations are overall robust, but “small and variable”^60^.

Nevertheless, to the best of our knowledge, the dimensions of valence and arousal were ignored in the studies employing causal interventions such as “the pen procedure”^20^, aiming to investigate the mechanism of sensorimotor simulation. This is not only important methodologically, but also theoretically because categorical tasks, such as assigning a stimulus to a category, rely on different cognitive skills than evaluative judgments on valence and arousal dimensions that can pick up broader changes in affective processes.

### Objectives and hypotheses of the present study

To sum up the theoretical and empirical background leading to the present study, numerous proponents of embodied cognition argue that facial mimicry (automatic imitation) is linked to sensorimotor simulation that supports recognition of perceived emotional stimuli^28,29,44^. Previous studies, whose aim was to investigate the causal role of facial responses in recognition of emotions, focused predominantly on visual modality, and a majority of them employed images of facial expressions as stimuli^20–24,46–49^. Only a handful of studies included different kinds of stimuli like bodily expressions^25^ or emotional vocalizations^51^. Moreover, this research focused primarily on distinct emotional categories (e.g., “happy”), neglecting dimensions of valence and arousal (but see^57^).

The aim of the present study was to investigate whether blocking facial mimicry influences recognition of emotional sounds, and the perception of their intensity, valence, and arousal. In our study, we addressed the problem of the generality of the effect of mimicry in two ways. First, we investigated whether mimicry is involved only in processing facial displays or whether it might be constitutive for processing emotional expressions associated with emotion concepts. In other words, we intended to investigate the causal involvement of mimicry as a reaction to the affective meaning of a stimulus, which is in line with the sensorimotor simulation account. Secondly, we aimed to investigate whether mimicry might be involved in the recognition process only or also in the evaluative judgments.

Participants listened to brief non-verbal vocalizations of four emotion categories (anger, disgust, fear, happiness) and neutral sounds. Stimuli were selected from the Montreal Affective Voices database (henceforth MAV^65^), designed to be analogous to the so-called “Ekman faces”^66,67^. In the mimicry blocking condition of our study, participants performed the task while holding the chopsticks horizontally between their teeth with their mouth closed around it. It is important to note that this frequently used manipulation leads to an elevated, constant, non-specific muscle activity in the lower part of the face. This is unlike some other manipulations, such as Botox, that prevent any activity in the region of the face^68^. In the control condition, participants held the chopsticks horizontally between their lips, in front of their teeth, keeping the lower face relaxed (modeled after Davis, Winkielman, and Coulson’s study^69^). After each stimulus presentation, participants evaluated the sound on seven visual analog scales regarding the extent to which the presented vocalization expressed anger, disgust, fear, and happiness, as well as its valence and arousal.

Biting chopsticks creates a constant muscular tension, and as such interferes with the dynamic response to the stimuli with the *zygomaticus major*, the facial muscle activated while smiling. Accordingly, we expected to find poorer emotion recognition, particularly of happy vocalizations, in the blocking cognition (in comparison to the control condition), analogous to that observed in the previous studies with facial images as stimuli^20–24^. Additionally, we examined whether this manipulation might change the evaluation of intensity, valence, and arousal of presented emotional vocalizations^57^.

## Method

### Participants

The study had 60 participants; one of them was excluded from the statistical analyses due to not following the experimental procedure. The mean age of the remaining experimental group was 25.2 year old (*SD* = 4.83; range 18–40), with 36 females and 23 males. All participants had normal or corrected-to-normal hearing, provided informed consent, and received 50 PLN (around 10 EUR) as a financial reward. All methods and procedures used in this study conformed to ethical guidelines for testing human participants. The study was approved by the Ethics Committee for Experimental Research at the Institute of Psychology, Jagiellonian University (decision: KE/27_2021).

### Materials

We used 50 brief sounds, which were nonverbal vocal expressions produced by humans of four basic emotions: anger, disgust, fear, happiness, and neutral sounds (vowel /ɑ/ sung in one note). Ten stimuli per each emotion category were used. The sounds were selected from the freely available MAV database^65^ and lasted approximately 1 s. As indicated by the validation provided by the database authors, the sounds were characterized by high decoding accuracy (ranging from 80 to 90%) apart from the fear expressing sounds (57% accuracy), which were often confused with the surprise expressions. Additionally, angry vocalizations had the lowest valence ratings, which was 16 on the 1–100 scale, followed by disgust (24), fear (24), neutral (47), and happy (85). As for the arousal, angry and fear expressing sounds were rated as the most arousing (both 72), followed by happy (57), disgust (36), and neutral (32) expressions.

### Procedure

Using facial electromyography, we checked with three participants whether mimicry blocking manipulation leads to the intended effect on the activity of the *zygomaticus major*. We expected that the blocking condition, which involved holding the chopsticks horizontally between the teeth with the mouth closed around it, would constantly increase muscle activity, thus preventing spontaneous mimicry, compared to the control condition, in which the muscles remained relaxed, allowing spontaneous mimicry.

Upon arrival at the laboratory, the experimenter presented participants with the general purpose of the study and explained the procedures. Then, participants gave their informed consent. Next, they went through the training, during which they learned the details of the task and practiced the elements of the experimental procedure.

The experimental procedure consisted of two blocks. In the experimental block (mimicry blocking condition), participants were asked to hold the chopsticks horizontally between their teeth with their mouths closed around them. In the control block, participants were asked to gently hold the chopsticks horizontally between their lips in front of their teeth keeping the lower face relaxed. This method was modeled on the study by Davis, Winkielman, and Coulson^69^. There were 25 sounds in each block, and five stimuli of each emotion category were randomly assigned to each block. The order of the blocks was assigned pseudo-randomly so that half of the participants began with the experimental block and the other half from the control block.

The participants’ task was to evaluate each sound on seven scales. The first five concerned the emotion category portrayed by a given sound. Participants were asked *To what extent the sound expressed the following emotion*? corresponding to all five emotion categories. The scales were presented in random order. The sixth scale concerned valence (*To what extent were the emotions expressed by the sound negative or positive?*) and the seventh arousal (*To what extent were the emotions expressed by the sound arousing?*). Those two scales were always presented in the same order.

The answers were given on an unmarked visual analog scale ranging from 1 to 100. Verbal labels were at the extremities with “not at all” on the left and “extremely” on the right for emotion category expressed and arousal scale items, and “very negative” on the left and “very positive” on the right for a valence scale. Each scale was displayed on a separate screen. The training and the experimental procedure were run using the PsychoPy software^70^. The sounds were played through the headphones (Philips SHP2500) and the sound volume was self-adjusted by the participants before the main task, during the training procedure to be audible yet comfortable. Overall, the task lasted up to 30 min.

## Results

### Emotion intensity

To analyze the influence of the mimicry blocking condition on the perceived extent to which presented sounds expressed portrayed emotions (emotion intensity), we conducted a repeated measures analysis of variance (RM-ANOVA) with within-subject factors of emotion (5) and blocking condition (2) using IBM SPSS Statistics 27. In all cases where the sphericity assumption has been violated, the results are reported with Huynh–Feldt correction. We used Bonferroni correction for pairwise comparisons. The analysis yielded significant effect of emotion (*F*(4,232) = 11.78, *p* < 0.001, partial η^2^ = 0.169), but no significant difference between blocking and control conditions (*F*(1,58) = 0.45, *p* = 0.504), or the interaction of those factors (*F*(4,232) = 1.04, *p* = 0.389).

Regarding the impact of emotion category, post hoc* t*-tests revealed that the relevant intensity rating was the lowest for angry sounds among all the categories (*M* = 62.12, *SE* = 2.04). Specifically, the score for angry vocalizations was lower than for disgust (*M* = 68.56, *SE* = 1.98, *p* = 0.031), fear (*M* = 76.5, *SE* = 1.97, *p* < 0.001), happiness (*M* = 76.32, *SE* = 2.55, *p* < 0.001), and neutral sounds (*M* = 77.54, *SE* = 2.36, *p* < 0.001). Happy vocalizations were rated as equally expressing the portrayed emotion as fear and disgust expressions, as well as neutral sounds. Intensity ratings for all portrayed emotions on each rating scale are presented in Table 1. Mean values and standard errors for emotion intensity are presented in Table 3. (See also Table S1 in the Supplementary Information for the intensity results divided into two mimicry blocking conditions).

**Table 1:** Mean intensity ratings for all portrayed emotions by a rating scale.

	Portrayed emotion				
Rating scale	Anger	Disgust	Fear	Happiness	Neutral
Anger	**62 (2)**	25 (1.9)	32 (2.4)	11 (1.7)	11 (1.6)
Disgust	36 (2.4)	**69 (2)**	41 (2.5)	12 (1.8)	17 (2.1)
Fear	45 (2.4)	20 (1.7)	**76 (2)**	9 (1.3)	12 (1.4)
Happiness	12 (1.3)	10 (1.2)	10 (1.2)	**76 (2.6)**	11 (1.4)
Neutral	19 (1.5)	29 (1.9)	17 (1.5)	23 (0.2)	**77 (2.4)**

Intensity was measured on a scale from 1 to 100. The table presents means and standard errors in brackets.

Values on the rating scale corresponding to the portrayed emotion are in [bold].

### Valence and arousal

There was a significant effect of mimicry blocking condition on valence (*F*(1,58) = 4.29, *p* = 0.043, partial η^2^ = 0.069) with a lower overall rating in the control condition (*M* = 37.13, *SE* = 0.89, vs. *M* = 38.91, *SE* = 0.82). The valence ratings significantly differed depending on emotion (*F*(4,232) = 260.12, *p* < 0.001, partial η^2^ = 0.82). The interaction of both conditions was not significant (*p* = 0.961).

The expressions of happiness (*M* = 71.26, *SE* = 2.24) were rated as the most positive among all five categories (all differences *p* < 0.001). Further, neutral sounds (*M* = 38.02, *SE* = 1.33) significantly differed from all emotional expressions (all differences *p* < 0.001). Disgust expressions (*M* = 26.95, *SE* = 1.03) were rated as more negative than happy and neutral sounds, but less negative than fear expressions (*M* = 22.07, *SE* = 1.15, *p* < 0.001), and equally negative to the angry vocalizations (*M* = 23.86, *SE* = 1.21, *p* = 0.081).

There were consistent differences between the blocking conditions in terms of valence. While the mimicry was disrupted, ratings were higher in all emotion categories. However, the biggest difference was observed for neutral stimuli. Mean values and standard errors for all emotion categories divided into two blocking conditions are presented in Table 2.

**Table 2:** Valence ratings. Mean values and standard errors for five emotion categories divided into two blocking conditions.

Portrayed emotion	Overall		Blocking condition		Control condition	
	*M*	*SE*	*M*	*SE*	*M*	*SE*
Anger	23.86	1.21	24.52	1.49	23.21	1.44
Disgust	26.95	1.03	27.82	1.35	25.98	1.32
Fear	22.07	1.15	23.0	1.29	21.14	1.52
Happiness	71.26	2.24	71.84	2.51	70.68	2.51
Neutral	45.96	0.74	47.26	0.68	44.67	1.04
Overall	38.02	1.33	38.91	0.82	37.13	0.89

Valence was measured on a scale from 1 to 100, with 1 being the most negative and 100 being the most positive.

Overall, arousal ratings did not differ between blocking conditions (*p* = 0.481). There was, however, a significant effect of emotion (*F*(4,232) = 173.84, *p* < 0.001, partial η^2^ = 0.75), while the interaction of blocking condition and emotion was not significant (*p* = 0.754).

Independent of the blocking condition, neutral sounds were rated as the least arousing (*M* = 23.29, *SE* = 1.87; all differences *p* < 0.001). Fear expressions (*M* = 69.56, *SE* = 1.76) were rated the highest, being rated as more arousing than the expressions of happiness (*M* = 61.54, *SE* = 1.83, *p* = 0.001) and disgust (*M* = 41.76, *SE* = 1.86, *p* < 0.001), and with marginal significance larger than the expressions of anger (*M* = 66.33, *SE* = 1.93, *p* = 0.054). The expressions of disgust were rated as less arousing than the expressions of anger, fear, and happiness (all differences *p* < 0.001). Mean values and standard errors for arousal ratings are presented in Table 3. (See also Table S2 in the Supplementary Information for the arousal ratings divided into two mimicry blocking conditions).

**Table 3:** Means and standard errors for emotion intensity, valence, arousal, and recognition.

Portrayed emotion	Emotion intensity		Valence		Arousal		Recognition	
	*M*	*SE*	*M*	*SE*	*M*	*SE*	*M*	*SE*
Anger	62.12	2.04	23.86	1.21	66.33	1.93	48.31	2.71
Disgust	68.56	1.98	26.95	1.03	41.76	1.86	65.25	2.6
Fear	76.5	1.97	22.07	1.15	69.56	1.76	68.47	2.52
Happiness	76.32	2.55	71.26	2.24	61.54	1.83	79.32	3.02
Neutral	77.54	2.36	45.96	0.74	23.29	1.87	76.27	2.88

All indices were measured on a scale from 1 to 100.

### Emotion recognition

Emotion recognition scores were based on intensity ratings. For each participant and each stimuli, if the highest emotion intensity was attributed to the scale relevant for the portrayed emotion, it was considered a *hit* (correct recognition). Based on averaged hits, we then conducted repeated measures ANOVA with blocking condition (2) and emotion (5) as within-subject factors.

Recognition rates varied depending on emotion category of the sound (*F*(4,232) = 24.035, *p* < 0.001, partial η^2^ = 0.293) but did not significantly differ between blocking conditions (*F*(1,58) = 0.208, *p* = 0.65). Additionally, the interaction of emotion and blocking condition was not significant (*F*(4,232) = 0.509, *p* = 0.729). Regarding the emotion category, angry vocalizations had lower recognition rates than any other category of vocalizations (all *p* < 0.001). Additionally, recognition rate of disgust was lower than that of happy (*p* = 0.002) and neutral sounds (*p* = 0.041). Fear vocalizations were recognized as accurately as vocalizations of disgust (*p* = 1.0), happiness (*p* = 0.102), and neutral sounds (*p* = 0.231). Mean values and standard errors for recognition scores are presented in Table 3. (See also Table S3 in the Supplementary Information for the recognition scores divided into two mimicry blocking conditions).

## Discussion

The main goal of the current study was to test whether disrupting spontaneous facial responses would influence the perception of emotional human vocalizations. The generalisation of the mimicry effect to auditory modality would suggest that sensorimotor processes engaged in face recognition are more broadly involved in recognizing emotional expressions. Moreover, we were interested in whether mimicry would be causally involved only in the recognition process or also in the more fundamental dimensional judgements. To this end, we focused on emotion recognition and the perception of stimuli’s intensity, valence, and arousal. We hypothesized that blocking spontaneous facial responses would lead to lower recognition scores for happy vocalization. Additionally, we expected that the mimicry blocking manipulation would also influence the evaluation of the intensity, valence, and arousal of emotional sounds.

Contrary to our predictions, disrupting the activity of the lower face muscles did not influence the recognition of vocalizations of neither happy, nor any other emotion category. Further, blocking mimicry did not significantly impact the perceived intensity and arousal of human affect vocalizations. Nevertheless, we observed a difference concerning judged valence between the conditions. When facial movements were disrupted, sounds were rated more positively than in the control condition; however, it is worth noting that the influence was minor and negative sounds still remained below the midpoint of the scale.

### Facial activity and recognition of emotional sounds

Numerous studies have shown that disrupting facial activity impacts the recognition of facial emotional expressions^20–25^. This effect is elucidated within the embodied simulation framework^28,29,44^. Since what is being simulated is not the expression on its own but an affective meaning of the expression^44,71^, we predicted that disrupting facial activity would also hinder the recognition of vocal portrayals of emotions.

Consistently, single studies have suggested that interfering with spontaneous mimicry affects the recognition of other modality expressions. Recently, Borgomaneri et al.^25^ found that blocking mimicry disrupts the recognition of both facial and whole-body expressions of emotions. Regarding the auditory channel, Hawk and colleagues’ study^51^ revealed that blocking spontaneous activity of lower-face muscle using the “pen procedure” (based on Strack, different from ours) influenced the time needed to spot the moment of transition between crying and laughing. However, their effects were non-intuitive and varied from research with transitions between facial expressions where blocking leads to slower, less accurate performance^21,68^. In the Hawk et al.’ study^51^, “blocked” participants performed the auditory task faster than participants able to move their faces freely.

Our study, however, did not corroborate these earlier findings. Interfering with the spontaneous activity of lower-face muscles did not have any selective or general impact on the accuracy of recognition of emotions portrayed with human affective vocalization. There might be several reasons for not observing the predicted effect. Somatosensory simulation, a postulated mechanism for conceptual processing^26,27^, consists of the selected brain and peripheral activations present during our past encounters with examples of a given category, including emotion concepts^71^. Peripheral activity, such as facial movements, does not have to be present at or involved in every instance of emotion recognition. Simulation is thought to be a flexible process, influenced by various factors^27,72^. It has been found to be of particular importance during the perception of expressions that are ambiguous or perceptually demanding^46,68^. In the case of our study, the vocalizations might have been too clear, hence requiring less mental processing. Further, certain social factors have been shown to be linked to facial responses to emotional expressions, including one’s motivation to bond and understand^15,73^. However, despite that some studies indicated several factors affecting mimicry, it is still a challenge for the embodied cognition account to provide a ground for drawing precise predictions as to when facial mimicry should occur and play a causal role in recognizing emotional expressions. This challenge is not unique to emotion categories or concepts but applies more generally to understanding ways to which more abstract concepts (including “anger” or “happiness”) relate to specific motor activity^36–38,43^ and translate into evaluative judgments^74^.

### Facial activity and valence perception

Interestingly, our study found that facial manipulation led to higher valence ratings in a non-category-specific manner, including neutral sounds. This is methodologically important and theoretically interesting and sheds light on the existing literature. Methodologically, it shows that our mimicry blocking manipulation (which involved slightly and constantly biting on the chopsticks) was effective. Theoretically, to the best of our knowledge, no study using this specific “pen procedure” to block spontaneous muscle activity has yet investigated valence, only recognition judgements. The only related study whose particular interest was on valence and arousal judgments was conducted by Hyniewska and Sato^57^. They presented participants with pictures of happy and angry facial expressions, and the participants’ task was to judge the expressions on valence and arousal scales while voluntarily contracting muscles following the task instruction (lower brows or raise cheeks). The authors found that raising the cheeks resulted in higher valence scores of both happy and angry expressions in comparison to the brow-lowering condition. Arousal was not influenced by the manipulation. Analogous to our experiment, the valence effect was not specific to one emotion category; namely, both happy and angry expressions received higher valence scores under the cheek-raising condition. The authors concluded that their results demonstrate the facial feedback effect^58,60,61^. The lack of neutral faces in Hyniewska and Sato’s study^57^ does not allow us to see whether the effect of facial manipulation would hold for neutral faces, as we observed for neutral stimuli in our experiment. Our study suggests that facial activity might not always be necessary for determining emotion category, however, it may play some role in evaluating expressions’ valence^63^.

### Evaluation of emotional vocalizations

All measured indices in this experiment varied depending on the emotion category represented by vocalizations. Negative emotions were characterized by the lowest recognizability. Specifically, angry vocalizations were attributed to the relevant category with the smallest degree of accuracy among all tested emotion categories. Vocalizations of disgust and fear had the next smallest degree of accuracy. Although angry vocalizations were rated the highest on a scale corresponding to the expressed emotion, they also received high scores on the scales for fear and disgust. Similarly, fearful vocalizations were often confused with anger and disgust expression. Compared to Belin et al.^65^, recognition scores of fearful vocalizations observed in our study were high. However, our study did not include surprise vocalizations, often confused with fearful sounds in the MAV database validation. Happy sounds were best identified (~ 80%). The highest recognition scores for the positive vocalizations in our study are not surprising since happy sounds was the only category characterized by positive valence. In the study by Belin et al.^65^, happy expressions were recognized accurately in 60% of cases; however, there they were often confused with another positive category: pleased vocalizations. In contrast, in Paquette et al.’s study^75^, the correct identification of happy vocalizations was nearly 100%. It is worth noting, however, that the evaluation from our study and the validations made by Belin et al.^65^, and Paquette et al.^75^, differed in several respects. First, they included a different number of emotion categories – from four in Paquette et al.’s validation, through five in our study, to nine in the MAV validation. Secondly, unlike Belin et al. and us, Paquette et al. used a forced-choice task with four options. Those two factors affected the level of difficulty. Thirdly, in the MAV validation procedure, participants assessed the actor’s emotion intensity, valence, and arousal. We focused on the evaluation of the stimuli. Therefore, observed discrepancies are likely due to methodological differences, and direct comparisons should be made with caution. The same applies to valence and arousal ratings.

Emotion recognition was based on intensity scores, and thus, the intensity ratings follow a similar, yet not identical, pattern. In the line of the recognition scores, angry vocalizations were rated the lowest; participants considered them to the lowest extent to express anger. There were no differences between fear, disgust, and happiness in this regard.

The expressions of happiness were considered the most positive, followed by neutral sounds, and disgusted, angry, and fearful vocalizations, respectively. Although this pattern is consistent with the one found by Belin et al.^65^, the results of our study are less extreme, e.g., 71 vs. 85 for happiness, 24 vs. 16 for anger. Arousal rating clearly depended on portrayed emotion. It was the highest for fearful and angry vocalizations, followed by happy expressions. Disgusted vocalizations were rated as the least arousing among all four emotion categories, while neutral vocalizations were considered the least arousing overall. This pattern of results is consistent with those previously described by Belin et al.^65^.

### Limitations and future directions

Drawing strong conclusions based on the results presented here is limited for several reasons. While we used continuous intensity rating scales for emotion categories to focus participants on dimensional aspects of emotion recognition, it is possible that the use of forced-choice (and thus categorical) task requiring time-pressured responses would reveal the effect of blocking facial mimicry in terms of accuracy and/or reaction times. Subsequent studies should investigate this possibility. Another limitation is related to the characteristics of the stimuli we used. It is known that simulation is stronger when stimuli are more ambiguous and more perceptually demanding^46,68^. The MAV’s vocalizations used in our study are perceptually clear and highly recognizable (especially happy vocalizations). It would be worthwhile to make the task more difficult by, for example, adding auditory noise to stimuli to make them more perceptually challenging or mixing vocalizations of different emotion categories in varying degrees to make them more ambiguous. The predicted effect of blocking facial mimicry could possibly be observed under these manipulations since they would force deeper conceptual processing of emotion categories. Furthermore, two recent studies revealed that emotional vocalizations taken from the MAV database are perceived as posed^76^ and evoke weaker facial reactions (measured with EMG) in comparison to authentic emotional vocalizations^77^. Less pronounced facial responses to the chosen set might have obscured the actual effect of facial mimicry blocking. The use of more ecologically valid stimuli might lead to revealing the effect. Finally, our study included only auditory stimuli; thus, direct comparisons with the recognition of facial stimuli is limited. This is important because our facial manipulation could lead to disruption of emotion recognition for faces (as seen in many previous studies), but only introduce a bias in the perception of non-facial stimuli, such as sounds or words.

Future studies may also investigate the differences between procedures used to “block mimicry”, as they are often treated as interchangeable but should lead to different effects. In some studies, participants were explicitly asked to voluntarily adopt certain facial configurations (e.g., resembling a smile), in other studies participants were put in a specific configuration by some method (e.g., pen in their mouth), yet other studies have attempted to fully disable the activity of the relevant muscles or their motor circuit (e.g., Botox, Transcranial Magnetic Stimulation; TMS). Specifically, our blocking procedure is based on the idea that a minor activation, such as creating a constant noise in the *zygomaticus* muscle, prevents it from selective dynamic responding to a positive stimulus^69,78^. Simultaneously, however, the constant activation of an emotion-relevant muscle creates a non-specific bias, as we, and others, have observed in valence ratings across all stimulus categories. In contrast, other “blocking” manipulations (like Botox) lead to a complete absence of responding from the relevant muscle^68^; the latter manipulation should not lead to any bias leading to non-specific enhancement or reduction in judgments of valence.

## Concluding remarks

The current study contributes to the continuing debate about the mechanisms underlying recognition and evaluation of important emotion stimuli, such as faces and emotional vocalizations. Our results suggest that somatosensory and motor feedback plays a role in the evaluation process, perhaps introducing a directional bias, but its role in the earlier stages of emotion recognition and categorization process may be minor or bound by important boundary conditions. This mirrors recent debates about the scope of claims in the embodied cognition literature, with many researchers arguing for a secondary role of somatosensory and motor processes, but perhaps not in a profound, constitutive way^33^. This distinction between stimulus recognition, stimulus categorization, and the subsequent stimulus evaluation is important for understanding at what processing stages, if any, somatosensory and motor processes are involved in cognitive and emotional processes.

## Supplementary Information

Supplementary Information.

## Data Availability

The data collected and analyzed during the current study are available from the corresponding author upon request.
