# Supplementary material for: Restricting movements of lower face leaves recognition of emotional vocalizations intact but introduces a valence positivity bias
Source: Sci Rep. 2022 Sep 27;12:16101. doi: 10.1038/s41598-022-18888-0 (PMC9515079; doi:10.1038/s41598-022-18888-0)
Supplement: Supplementary file 1 — Supplementary Information. [file 41598_2022_18888_MOESM1_ESM.pdf]

Supplementary Information to:
Restricting movements of lower face leaves recognition of emotional vocalizations intact but introduces a valence positivity bias

Kinga Wołoszyn, Mateusz Hohol, Michał Kuniecki, Piotr Winkielman

Table S1. Intensity ratings. Mean values and standard errors for five emotion categories divided into two blocking conditions.

	Overall		Blocking condition		Control condition	
Portrayed emotion	*M*	*SE*	*M*	*SE*	*M*	*SE*
Anger	62.12	2.04	62.5	2.56	61.73	2.41
Disgust	68.56	1.98	66.29	2.65	70.82	2.11
Fear	76.5	1.97	76.38	2.08	76.62	3.36
Happiness	76.32	2.55	76.91	2.7	75.73	2.79
Neutral	77.54	2.36	76.75	2.6	78.32	2.65
Overall	72.21	1.33	71.77	1.46	72.64	1.51

Note. The intensity was measured on a scale from 1 to 100. 1 indicated that the sound expressed the portrayed emotion to the lowest degree and 100 indicated that the sound expressed the portrayed emotion to the highest degree.

Table S2. Arousal ratings. Mean values and standard errors for five emotion categories divided into two blocking conditions.

	Overall		Blocking condition		Control condition	
Portrayed emotion	*M*	*SE*	*M*	*SE*	*M*	*SE*
Anger	66.33	1.93	65.16	2.21	67.5	2.21
Disgust	41.76	1.86	41	2.1	42.52	2.01
Fear	69.56	1.76	69	2.17	70.12	1.81
Happiness	61.54	1.83	61.65	2.21	61.43	2.11
Neutral	23.29	1.87	23.7	2.34	22.88	1.96
Overall	52.5	1.29	52.1	1.52	52.9	1.3

Note. Arousal was measured on a scale from 1 to 100. 1 indicated that the sound was arousing to the highest degree and 100 that the sound was arousing to the highest degree.

Table S3. Recognition scores. Mean values and standard errors for five emotion categories divided into two blocking conditions.

	Overall		Blocking condition		Control condition	
Portrayed emotion	*M*	*SE*	*M*	*SE*	*M*	*SE*
Anger	48.31	2.71	48.1	3.2	48.5	3
Disgust	65.25	2.6	63.4	3.6	67.1	2.8
Fear	68.47	2.52	68.5	2.6	68.5	3.5
Happiness	79.32	3.02	80	3.3	78.3	3.4
Neutral	76.27	2.88	75.3	3.4	78.3	3.4
Overall	67.5	1.6	67.1	1.7	67.9	2

Note. Recognition was measured on a scale from 1 to 100. The higher the score, the most accurately the sounds were recognized.
